# Diagnosis of C5 segmental vertebral artery using magnetic resonance angiography

**DOI:** 10.1007/s00276-025-03640-w

**Published:** 2025-04-30

**Authors:** Kenji Takata, Midori Ueda, Kiyotaka Takeuchi, Hideaki Komiya, Daisuke Yoshikawa, Satomi Kanai, Tasuku Wakabayashi, Ayaki Kitano, Mariko Toyooka, Toyohiko Sakai, Tetsuya Tsujikawa

**Affiliations:** Department of Radiology, Fukui University, Fukui, Japan

**Keywords:** Vertebral artery, Vertebral artery variation, C5 segmental VA, Magnetic resonance angiography

## Abstract

**Purpose:**

The vertebral artery (VA) typically enters the subarachnoid space at the atlanto-occipital region. However, segmental variations can occur, with the VA entering the spinal canal at atypical levels. While a C3 segmental VA has been reported, no prior studies describe a C5 segmental VA. This case represents the first documented occurrence of this anomaly.

**Methods:**

An 8-year-old girl underwent brain magnetic resonance imaging for headache screening, which incidentally revealed an abnormal VA course.

**Results:**

Imaging revealed the absence of the left VA at the proximal V2 segment. Instead, a radiculomedullary artery at C4/5 entered the spinal canal and contributed to the formation of the anterior spinal artery (ASA), which ascended along the spinal cord. The right VA appeared normal; however, a radiculomedullary artery at the C3/4 level was identified, joining the contralateral radiculomedullary artery at the C1 level to form the ASA. Additionally, bilateral accessory middle cerebral arteries were observed. No clear association was found between this anomaly and the patient’s headache, and she remained under observation.

**Conclusion:**

This anomaly may result from persistence of the fifth intersegmental artery. The vascular course resembled collateral circulation observed in acquired VA occlusion. Given its proximity to the spinal cord, potential risks include ischemic complications and spinal cord compression. This case highlights the importance of accurate imaging and careful surgical planning. Further studies on these rare vascular anomalies will enhance our understanding of VA variations and their clinical significance.

## Introduction

The V3 segment of the vertebral artery (VA) typically enters the subarachnoid space via the atlanto-occipital space. However, certain cases exhibit segmental variations in which the artery enters the spinal canal at an alternative vertebral level. One such variation is the C3 segmental VA, where the artery enters the spinal canal at the C2/3 level, as documented in the literature [1, 3, 8].

Previous studies have emphasized the clinical significance of the C3 segmental VA, particularly concerning cervical spine surgery and the potential risks of arterial injury or dissection. Understanding these anatomical variations is essential for surgical planning and vascular evaluations [1, 3, 8].

Here, we report a rare case of a C5 segmental VA, an anatomical variation not previously documented in the literature. This report provides insights into VA variations and explores their potential clinical implications.

## Case report

The patient, an 8-year-old girl, presented with a chief complaint of headache. She was born preterm with low birth weight; however, her subsequent development was uneventful, showing no anomalies.

**Fig. 1:**
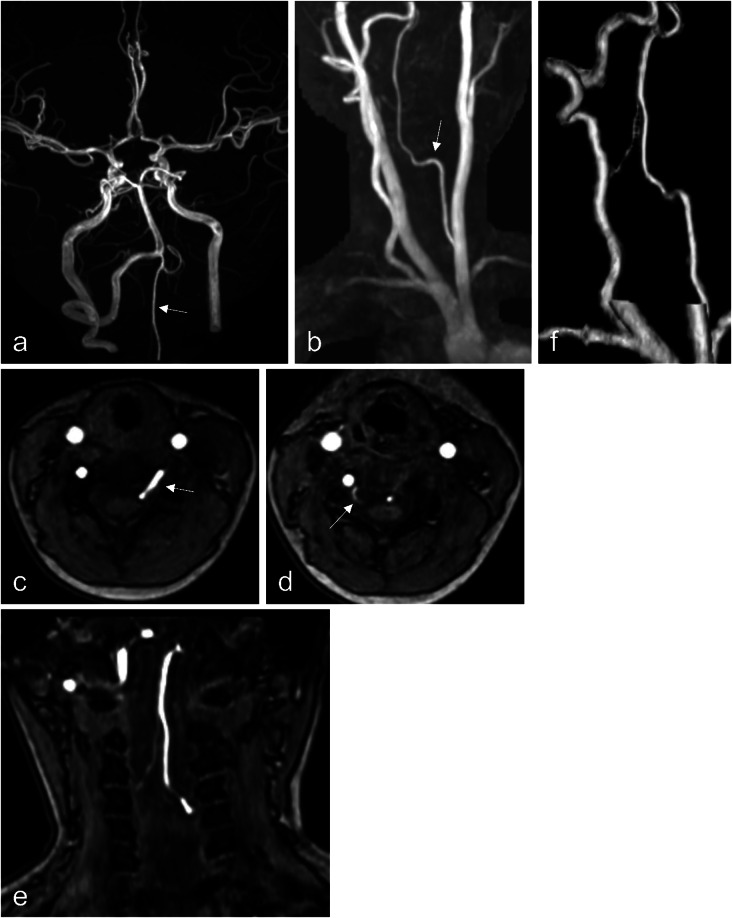
Head and neck MRA images using a 3 T magnetic resonance imaging scanner. **a**: Anteroposterior projection of a maximum intensity projection (MIP) image showing the absence of a normal left VA course with visualization of the developed anterior spinal artery as a collateral pathway (arrow). **b**: Lateral MIP projection showing the ectopic course of the left vertebral artery (arrow). **c**: MRA source image at the level of the C4–C5 vertebrae shows the entry of the left VA into the spinal canal via the C4–C5 intervertebral foramen (arrow). **d**: MRA source image at the level of the C3–C4 vertebrae shows the entry of the small branch (radiculomedullary artery) originating from the right vertebral artery into the spinal canal via the C3–C4 intervertebral foramen (arrow). **e**: Coronal section of the MR angiographic source image. Two blood vessels ascending within the spinal canal converge at the C1 level to form an anterior spinal artery. **f**: A volume-rendered image focusing on the vertebral artery is shown, with the above vascular anatomy clearly defined

She underwent a brain MRI for screening purposes, which revealed an abnormal course of the left VA (Fig. 1). The left VA was absent at the proximal V2 segment, and a well-developed radiculomedullary artery coursed through the C4/5 intervertebral foramen, serving as a collateral pathway.

The right VA appeared normal; however, a radiculomedullary artery was observed at the C3/4 level. This artery joined the contralateral radiculomedullary artery at the anterior surface of the spinal cord at the C1 level, forming the anterior spinal artery (ASA). The ASA ascended and formed the distal V4 segment of the left VA, which subsequently merged with the right VA to create the basilar artery (BA).

Additionally, the patient had an accessory middle cerebral artery originating proximally on the right side and distally on the left side.

There was no evident connection between these vascular anomalies and the patient’s headache, so she was placed under observation.

## Discussion

C5 segmental VA anomaly is exceptionally rare, with significant clinical and surgical implications. The discussion can benefit from insights presented in the previously cited articles on C3 segmental VA and its associated anomalies.

### Frequency and embryology of segmental VAs

Segmental VA is a rare variation of VA, with its embryological development associated with the intersegmental arteries [4]. It arises from the persistence of one of these arteries during fetal development, resulting in an abnormal course. In normal embryology, the final intersegmental artery becomes the subclavian artery, while the other intersegmental arteries regress. However, the persistence of an intersegmental artery between C5 and C6 can lead to the formation of a C5 segmental VA.

Segmental VAs, such as C2 and C3, are generally rare. The prevalence of C2 segmental VA is approximately 3.2% [7]. In contrast, C3 segmental VA has been documented only a few times, emphasizing its rarity [1, 3, 8]. C5 segmental VA is even more uncommon, with no similar cases reported to date.

The C3 segmental VA develops as it curves medially below the articular process of C2 and penetrates the dura through the C2/3 neural foramen due to the persistence of the third intersegmental artery. A similar mechanism is involved in the development of the C5 segmental VA, where the fifth intersegmental artery remains without regression, determining the observed vascular pathway in this instance.

**Fig. 2:**
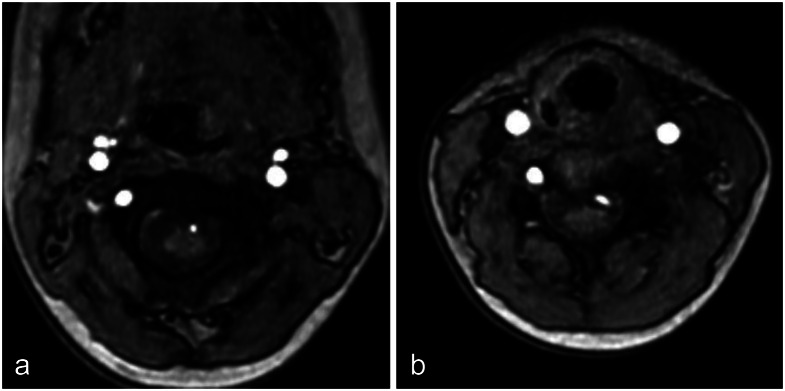
Axial source images of cervical MRA using a 3 T magnetic resonance imaging scanner. **a**: Axial image at the level of the C2 vertebra shows absence of the left transverse foramen. **b**: Axial image at the level of the C4 vertebra also shows absence of the left transverse foramen. These findings support a congenital defect of the left vertebral artery pathway

Zhang et al. [9] reported that such a vascular course can occur. They stated that in cases where the VA is occluded due to acquired factors such as atherosclerosis or arterial dissection, the radiculomedullary artery and ASA may develop as collateral pathways. In this particular instance, the VA penetrated the spinal canal at the C4/5 level, exhibiting a vascular pattern similar to collateral circulation observed in acquired VA occlusions. However, this case involved a congenitally healthy girl, making an acquired etiology unlikely. Although CT was not performed due to concerns about radiation exposure, source images from the cervical MRA revealed absence of the transverse foramina on the left side from C2 to C4, supporting the hypothesis of a congenital origin (Fig. 2). It is therefore postulated that failure in the development of the proximal V2 segment during embryogenesis led to the anomalous vascular trajectory entering the spinal canal at the C4/5 level.

### Formation of the ASA at the C1 level

The ASA forms through the fusion of the left and right ventral longitudinal neural arteries at the midline. Lack of fusion in the ASA can result in duplication or fenestration, which are not classified as anatomical anomalies but are instead linked to the incomplete maturation of the ventral longitudinal neural arteries [2].

In this case, the vessels entering the spinal canal from the left and right vertebral arteries did not immediately converge; rather, they merged at the C1 level to create an ASA. This vascular configuration is believed to result from the incomplete maturation of the ventral longitudinal neural arteries, suggesting the entry of radiculomedullary arteries into the spinal canal.

### Symptoms

Segmental variations in the VAs, particularly the C3 and C5 types, present a significant risk for spinal cord compression. These segmental VAs exhibit an anomalous course situated proximal to the spinal cord and nerve roots, which can lead to pulsatile compression. Bilateral segmental VAs can induce spinal cord compression, potentially causing myelopathy, which is a serious concern [6]. The symptoms include neck pain, sensory and motor dysfunction, and muscle weakness in the upper limbs.

Additionally, anomalies during the course of VA are associated with Bow Hunter’s syndrome. In this condition, VA stenosis or occlusion occurs temporarily because of neck rotation, resulting in ischemic symptoms [5]. Rastogi V et al. reported that specific neck movements can impact VA hemodynamics, resulting in dizziness and transient ischemic attacks [5].

In our case, the C5 segmental VA may have caused transient blood flow disturbances through a mechanism similar to that of Bow Hunter’s syndrome. Although no symptoms were observed at present, close follow-up is necessary to monitor for potential ischemic events.

### Surgical considerations

The presence of C5 segmental VA may elevate the risk of vascular injury during surgery, emphasizing the necessity of detailed preoperative imaging assessments. Precise identification of an anomalous vascular course is crucial for establishing a safe surgical plan [3]. These vascular anomalies can be diagnosed using advanced imaging modalities, such as computed tomography (CT) angiography, magnetic resonance angiography (MRA), and MRI [8]. Similar to C3 segmental VA, where a posterior approach is commonly selected, considering the anatomical features of C5 segmental VA is essential when determining the suitable surgical approach.

### Personal perspective

This case concerned my oldest daughter. I informed her that she has a rare vascular anatomy, and she received this information with interest. In her daily life, she practices cheer dances and can perform flexible techniques without any issues. At present, there are no indications of ischemia or neurological symptoms from vascular anomalies, and we anticipate no future occurrences of such effects.

## Conclusion

C5 segmental VA is an extremely rare variant that holds significance in elucidating the developmental mechanism of VA and its surgical implications. Precise diagnosis and surgical planning are essential, with imaging modalities such as CT angiography and MRA playing crucial roles. Surgical interventions also require meticulous planning and execution. Additional case studies are crucial for enhancing comprehension of these rare anatomical variances and their clinical significance.

## Data Availability

No datasets were generated or analysed during the current study.
